# A strategy to study pathway cross-talks of cells under repetitive exposure to stimuli

**DOI:** 10.1186/1752-0509-6-S3-S6

**Published:** 2012-12-17

**Authors:** Yan Fu, Xiaoshan Jiang, Hang Zhang, Jianhua Xing

**Affiliations:** 1 Department of Biological Sciences, Virginia Tech, Blacksburg, VA 24060, USA; 2 Interdisciplinary PhD Program of Genetics, Bioinformatics and Computational Biology, Virginia Tech, Blacksburg, VA 24060, USA

## Abstract

**Background:**

Cells are subject to fluctuating and multiple stimuli in their natural environment. The signaling pathways often crosstalk to each other and give rise to complex nonlinear dynamics. Specifically repetitive exposure of a cell to a same stimulus sometime leads to augmented cellular responses. Examples are amplified proinflammatory responses of innate immune cells pretreated with a sub-threshold then a high dose of endotoxin or cytokine stimulation. This phenomenon, called priming effect in the literature, has important pathological and clinical significances.

**Results:**

In a previous study, we enumerated possible mechanisms for priming using a three-node network model. The analysis uncovered three mechanisms. Based on the results, in this work we developed a straightforward procedure to identify molecular candidates contributing to the priming effect and the corresponding mechanisms. The procedure involves time course measurements, e.g., gene expression levels, or protein activities under low, high, and low + high dose of stimulant, then computational analysis of the dynamics patterns, and identification of functional roles in the context of the regulatory network. We applied the procedure to a set of published microarray data on interferon-γ-mediated priming effect of human macrophages. The analysis identified a number of network motifs possibly contributing to Interferon-γ priming. A further detailed mathematical model analysis further reveals how combination of different mechanisms leads to the priming effect.

**Conclusions:**

One may perform systematic screening using the proposed procedure combining with high throughput measurements, at both transcriptome and proteome levels. It is applicable to various priming phenomena.

## Background

A cell needs to constantly sense and response to various signals from both external and internal environments. The requirement on generating appropriate response to specific signals forces cells to develop a complex signaling network that often involves multiple highly intertwined signaling pathways [1-3]. It becomes increasingly clear that pathway cross-talks play critical roles in cellular signaling and decision making process [4]. For example, cross-talks may increase the nonlinearity in the signaling network, resulting in various synergistic and antagonistic effects in cellular responses [5-8]. A nonlinear response refers to the cellular response to multiple different stimuli, or repetitive stimulus that is not simply the sum of responses to each individual stimulus. Cells *in vivo *are constantly exposed to a variety of stimulus with fluctuating concentration. Therefore it is of great importance to study how cells utilize complex pathway cross-talks to generate appropriate response or make correct decision to multiple or repetitive stimulus. Pharmaceutically, it is also a common treatment strategy to use combinations of multiple drugs simultaneously in order to generate synergistic effect [8,9]. Therefore, the nonlinear phenomena due to pathway cross-talks have important physiological and clinical significances.

**Figure 1:**
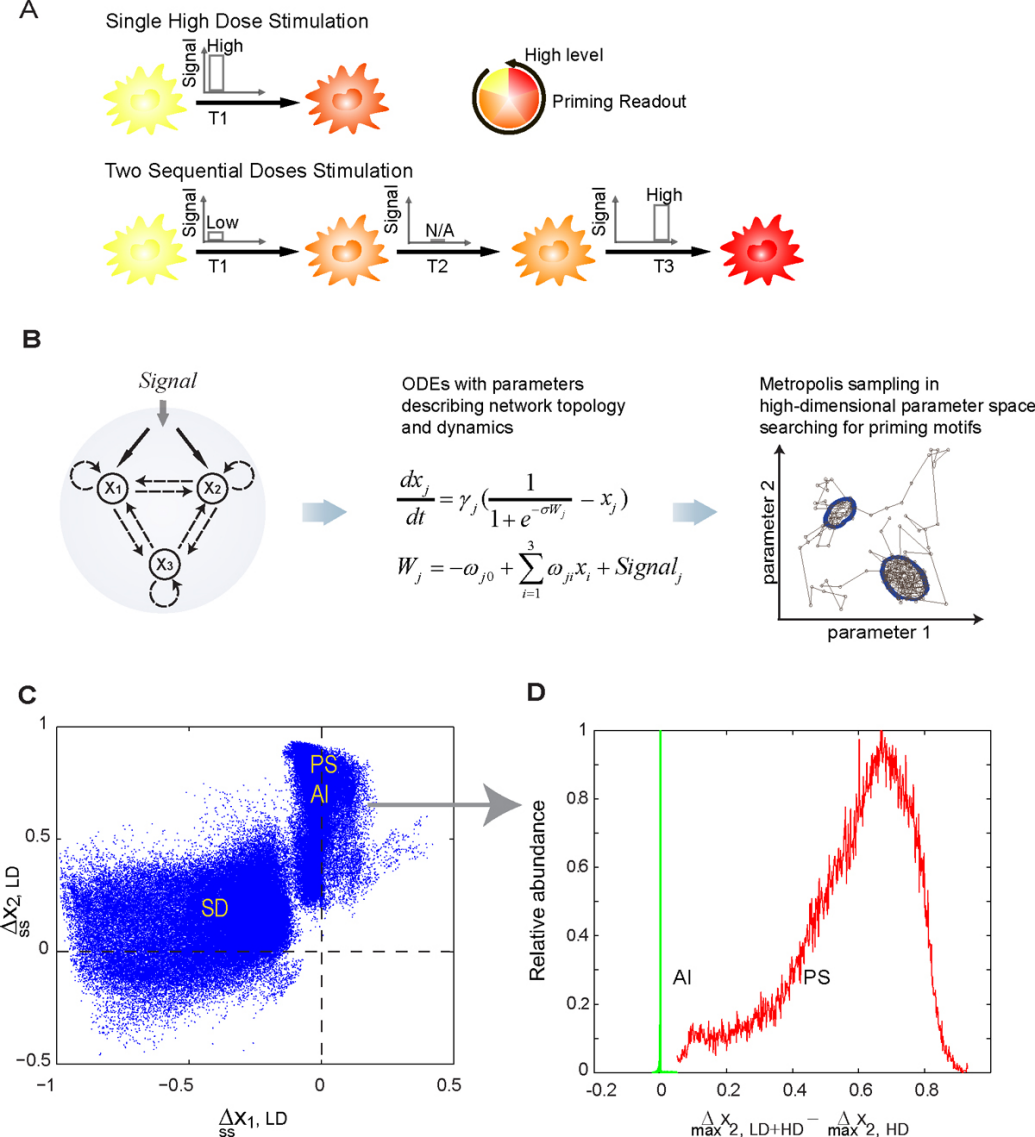
**Summary of theoretical analysis**. A) Schematic illustration of the experimental procedure inducing priming effect. B) An abstract three-node model was chosen to represent that the stimulus can activate two parallel pathways (through x_1 _and x_2_) which converge to the monitored readout (x_3_). A set of corresponding ordinary differential equations (ODEs) were constructed, and a Metropolis sampling algorithm was used to search for parameter sets giving the priming effect in the high-dimensional parameter space. C) Computational studies show that the parameter sets leading to priming naturally divide into two regions, corresponding to different priming mechanisms. $\underset{ss}{\Delta }x_{1,LD}$ ($\underset{ss}{\Delta }x_{2,LD}$): change of x_1 _(x_2_) level at the end of LD treatment period compared to those of untreated cells. D) The right region in C) can be further discriminated according to the sample abundance distribution (relative to the maximum within each group) of $\underset{\text{max}}{\Delta }x_{2,LD+HD}-\underset{\text{max}}{\Delta }x_{2,LD}$ (difference between the maximum level of x_2 _during the HD treatment period after LD pretreatment and that of x_2 _without LD pretreatment), suggesting overall three priming mechanisms. Panels A and B are adapted from [25].

In this work we focus on cellular priming effect (also called preconditioning and sensitization) which refers to a well-observed phenomenon that after being treated with a seemingly negligible concentration of stimulus, a cell may launch amplified responses upon a second exposure to the same stimulus at higher concentration [10-12]. The priming effect reflects the nonlinear characteristics of the system in that the cellular response to repetitive stimuli is stronger than the sum of that to individual low dose and high dose stimulation. Since the cellular response to the low dose stimulation is negligible, in experimental practice one usually approximates the above sum by the cellular response under the high dose stimulation only. Two such examples are lipopolysaccharide-mediated (LPS) and Interferon-γ-mediated (IFN-γ) priming effects observed in innate immune cells such as monocytes and macrophages [11,13]. For example, LPS is the pathogen-associated molecular pattern (PAMP) expressed on the outer membrane of gram-negative bacteria. Several *in vitro *studies have reported that low dose LPS (e.g., 0.05-1 µg/L) can prime macrophages for an augmented pro-inflammatory cytokine production under high dose LPS (10-100 µg/L) [10,12-15]. Clinically, evidence relates this LPS-mediated priming phenomenon to low-grade metabolic endotoxemia, which is defined as an elevated but physiological LPS concentration in the blood, resulting in a higher incidence of insulin resistance, diabetes and atherosclerosis [16-21]. Similarly, a sub-activating dose of IFN-γ (e.g., 0.05-0.15 µg/L) is able to prime macrophages for an enhanced activity of signal transducer and activator of transcription 1 (STAT1) under an activating dose of IFN-γ (e.g., 0.5-5 µg/L) (Figure 1A). As a consequence, the expression of a number of genes regulated by STAT1 are also increased, including IFN regulatory factor 1 (IRF-1) and inducible protein-10 (IP-10). Since IFN-γ plays a crucial role in interfering viral replications and promoting apoptosis of infected cells, abnormality in IFN-γ production can lead to severe consequences in the immune system [22]. The sensitization of IFN-γ signaling also correlates with several immune system malfunctions and diseases, such as rheumatoid arthritis, hepatitis and multiple sclerosis [22-24]. Hu et al., first investigated the molecular mechanisms of IFN-γ-mediated priming effect and reasoned that an elevated expression of STAT1 by low dose pretreatment was responsible for the induction of priming effect [11]. However, other molecular mechanisms may also exist.

In the previous study, we applied a computational analysis to enumerate all possible network motifs that are able to induce priming effect in a generic three-node regulatory network. Strikingly, we found that the *in silico *discovered priming motifs naturally fall into three priming mechanisms. Based on the finding, the main purpose of this study is to design and apply a general combined experiment and computation strategy to search for molecular candidates contributing to the priming effect for a given stimulus. The remaining part of the paper is organized as follows. First we summarize the main results of our first study, and outline the strategy. Then we demonstrate how to apply the strategy to analyze a set of published microarray data on IFN-γ-mediated priming effect. Next we show further analysis on a detailed ordinary differential equation based model.

## Results and discussions

### Computational analysis suggests basic priming mechanisms

In the first paper [25], we enumerated all possible network structures and kinetics that are able to induce priming effect with a generic three-node model (Figure 1B). The three-node model represents the minimal abstraction of the two cross-talking pathways (e.g., MyD88-dependent and -independent branches of Toll-like receptor 4 (TLR4) signaling pathway). Each node in the model can either positively or negatively regulate the activity of the other nodes or itself. We simulated the dynamics with a set of nonlinear ordinary differential equations with 14 variable parameters. Through a two-stage Metropolis algorithm, we analyzed the dynamical behavior of over 1.5 × 10^5 ^different networks that can generate priming effect. Here we refer to priming effect as a set of dose-response behaviors: (1) A single low dose stimulant (LD) cannot activate the readout x_3 _(< 0.1 in a reduced unit with 1 the maximum induction). (2) A single high dose stimulant (HD) can activate x_3_. (3) Sequential stimulation with LD first followed by HD (LD+HD) can activate x_3 _to a maximum level that is at least 50% higher than that under HD alone.

As shown in Figure 1C, the parameter sets leading to priming effect clearly cluster into two regions, in terms of the change in the two regulators, x_1 _and x_2_, at the end of LD pretreatment ($\underset{ss}{\Delta }{x_{i}}_{,LD}$, *i *= 1,2). Data in the left region locate approximately along the negative side of x-axis, that is, a LD pretreatment decreases x_1 _in this region (i.e., $\underset{ss}{\Delta }{x_{1}}_{,LD}<-\delta <0$, with an arbitrarily chosen cutoff δ=0.1to account for possible experimental resolution). Notice x_2 _in this region spread out vertically, that is, x_2 _can either increase or decrease to some extent under LD pretreatment. Based on this observation, we want to find out any possible constraint on x_2 _in this region. To do this, we plotted the distribution of the difference between the maximum response of x_2 _under LD+HD and that under HD alone. We found that x_2 _from this region can be either HD-responsive or LD-responsive, but with a constraint that the maximum expression under LD+HD makes no difference with that under HD alone (i.e., $\underset{\text{max}}{\Delta }x_{2}{,}_{LD+HD}\approx \underset{\text{max}}{\Delta }x_{2}{,}_{HD}$) [see Additional file 1]. On the other hand, the data in the right region demonstrate a significant increase in x_2_, but not x_1_, after LD pretreatment (Figure 1C) (i.e., $\underset{ss}{\Delta }{x_{2}}_{,LD}>\delta$). The maximum expression of x_1 _under LD+HD makes no difference with that under HD alone (i.e.,$\underset{\text{max}}{\Delta }x_{1}{,}_{LD+HD}\approx \underset{\text{max}}{\Delta }x_{1}{,}_{HD}$) [see Additional file 1]. However, this overlapped region can be further separated into two sub-groups, pathway synergy (PS) and activator induction (AI), if plotted against another experimentally measurable quantity: the difference in the maximum level of x_2 _under LD+HD *vs *under HD (Figure 1D). It is obvious that the data from the red group, but not the green group, shows a significant increase in the maximum level of x_2 _under LD+HD compared to that under HD alone (i.e., $\underset{\text{max}}{\Delta }x_{2}{,}_{LD+HD}-\underset{\text{max}}{\Delta }x_{2}{,}_{HD}>0$) (Figure 1D).

**Figure 2:**
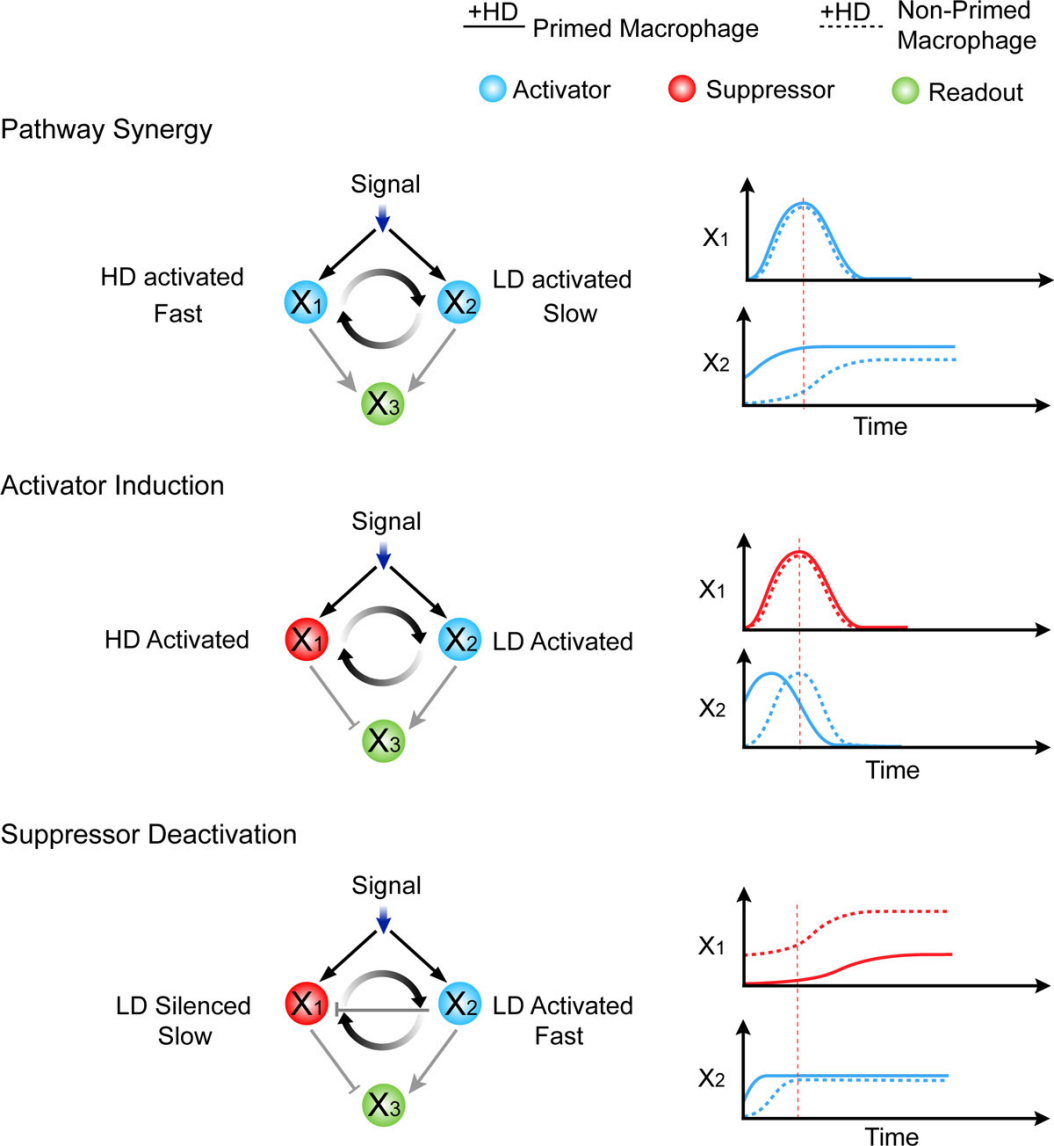
**Schematic illustration of the three *in silico *found priming mechanisms**. The left column shows the basic topological requirement identified in the corresponding priming dataset generated in the theoretical analysis. The right column shows the typical time course of each priming mechanism.

Further statistical analysis on network topologies reveals that data from each priming group shares a unique network structure (Figure 2, left column). For example, x_1 _in the left region in Figure 1C is identified as an inhibitor to the readout x_3_. Since x_1 _is decreased by LD, we therefore named this region "Suppressor Deactivation" (SD). Similarly, x_2 _in right region in Figure 1C is found to be an activator to x_3_. Based on the fact that the data in this region can be further differentiated in terms of differential dose-response$\underset{\text{max}}{\Delta }x_{2}{,}_{LD+HD}-\underset{\text{max}}{\Delta }x_{2}{,}_{HD}$, we further named them "Pathway Synergy" (PS, denoted in red) and "Activator Induction" (AI, denoted in green), respectively (Figure 1D).

The physics underlying the three priming mechanisms turns out to be simple and beyond the current three-node model [25]. For Pathway Synergy, both of the two pathways activate the priming readout x_3_, but one has a fast time scale and a high activation threshold while another one has a slow time scale and a low activation threshold. When given a single HD stimulation, the regulation on x_3 _from the two pathways is temporally separated. A LD pretreatment brings forward the slow pathway so that the two pathways can achieve a transient synergy to boost the production of x_3 _(Figure 2). Similarly, for Activator Induction and Suppressor Deactivation, a LD pretreatment separates the two originally temporally overlapping but antagonistic pathways by either advancing the activator or delaying the suppressor (Figure 2).

**Figure 3:**
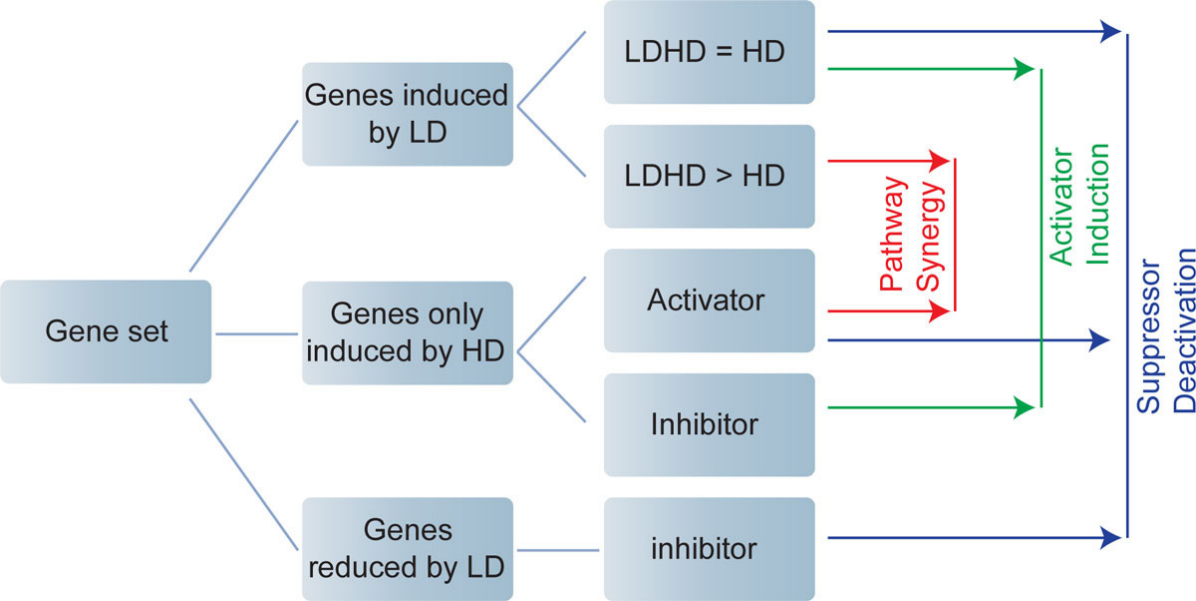
**Proposed procedure of microarray analysis for identifying candidate genes under different priming mechanism**. LD: low dose stimulation; HD: high dose stimulation. Second column: Genes are grouped according to their behaviors under LD and HD, respectively. Third column: genes are further sub-grouped according to the differential expression under LD+HD or under HD alone. Genes that can only be induced by HD are further differentiated according to their regulatory behavior (e.g. activator or inhibitor) to the readout gene. Fourth column: combinations of gene from different sub-groups reveal potential priming motifs (x_1 _and x_2 _in Figure 2).

Since each priming mechanism highlights unique topological and dynamical characteristics, we propose that one can utilize this important information to guide microarray analysis on identifying groups of candidate genes that contribute to priming effect. The computational result in Figure 1C and 1D actually suggests a simple procedure to this purpose. The analyzing procedure is summarized as follows (also see Figure 3):

1. Record the time course of the cellular response under single LD, single HD, and LD+HD, respectively.

2. Identify the priming readout genes as those with higher response to LD+HD than HD, but with no significant response to LD.

3. Identify the genes induced or reduced by LD (LD-responsive genes), and those responding to HD only (HD-responsive genes).

4. Construct the interaction network through integrating the available experimental results, and available databases. Examine the identified genes in the context of the network regulations and identify the corresponding molecular mechanisms for priming they potentially contribute to:

• Pathway Synergy: (1) LD-responsive genes (with the expression under LD+HD higher than that under HD alone) and (2) HD-responsive genes; (3) both activate a downstream readout gene.

• Activator Induction: (1) LD-responsive genes (with the expression under LD+HD similar to that under HD alone) and (2) HD-responsive genes; (3) the LD-responsive gene activates while the HD-responsive gene inhibits a downstream readout gene.

• Suppressor Activation: (1) LD-reduced genes and (2) LD/HD-responsive genes (with the expression under LD+HD similar to that under HD alone); (3) the LD-reduced gene inhibits while the LD/HD-responsive gene activates a downstream readout gene.

### Microarray data analysis predicts possible candidates involved in the induction of IFN-γ-mediated priming effect

**Figure 4:**
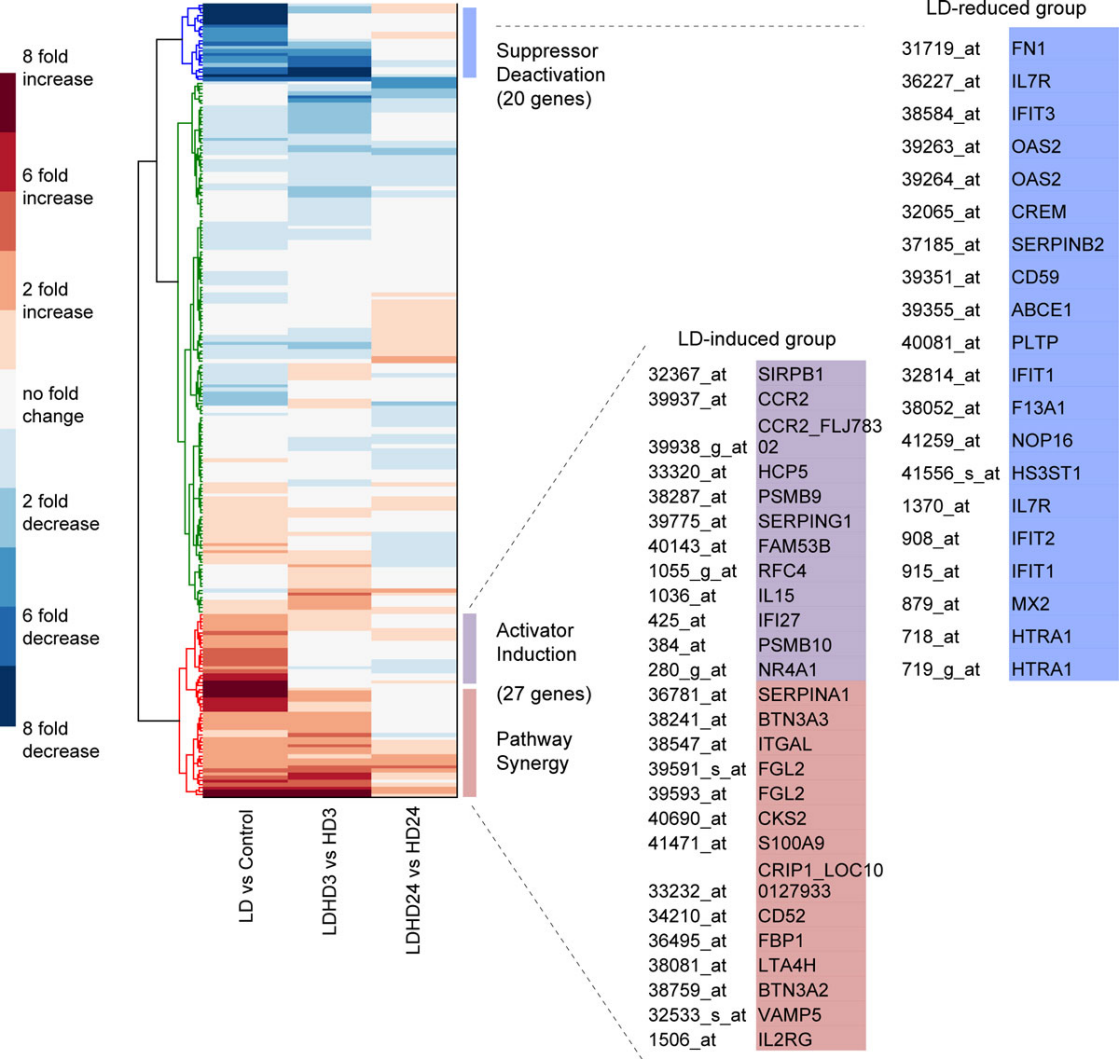
**Analysis of the microarray data (GEO, accession number: GDS1365)**. Hierarchical clustering of the gene expression profiles over 225 genes. The left, middle and the right columns denote the fold change under LD *vs *Control, LD+HD *vs *HD (3 hr), and LD+HD *vs *HD (24 hr), respectively. Genes that are statistically increased and decreased by LD are listed on the right. These genes are grouped into different priming mechanisms according to the guideline shown in Figure 3.

In this section, we focus on the microarray data on IFN-γ by Hu et al. [26] in order to demonstrate the proposed analyzing procedure. This is the only set of data we found from the microarray database Gene Expression Omnibus that satisfies the requirement in the above discussed procedure. After two steps of data processing (see Methods for details), we found 225 genes demonstrating non-trivial dynamics (i.e., with statistically significant change under at least one condition, see Methods for details). They form the subjects of our analysis. Hierarchical clustering of these genes shows that the majority of them do not show statistically significant change (by ≥ 2 fold) under LD (Figure 4). However, we found that 27 genes are significantly increased (by ≥ 2 fold) by LD, and 20 significantly decreased (by ≥ 2 fold) by LD (Figure 4, the probe names and gene symbols are listed on the right). Based on the proposed analyzing procedure, these genes constitute the candidate regulators for different priming mechanisms (Figure 3). These genes will then be subject to further analysis, such as examining them in the context of the regulatory network (discussed below). Moreover, since the level of the LD-responsive regulator in PS mechanism is dramatically increased under LD+HD than under HD alone, while the corresponding regulator in AI barely shows any difference (Figure 1D), these 27 LD-responsive genes can be further sub-grouped into either PS or AI category based on their expression profiles accordingly (i.e., $\underset{\text{max}}{\Delta }x_{i,LD+HD}-\underset{\text{max}}{\Delta }x_{i,HD}$).

**Figure 5:**
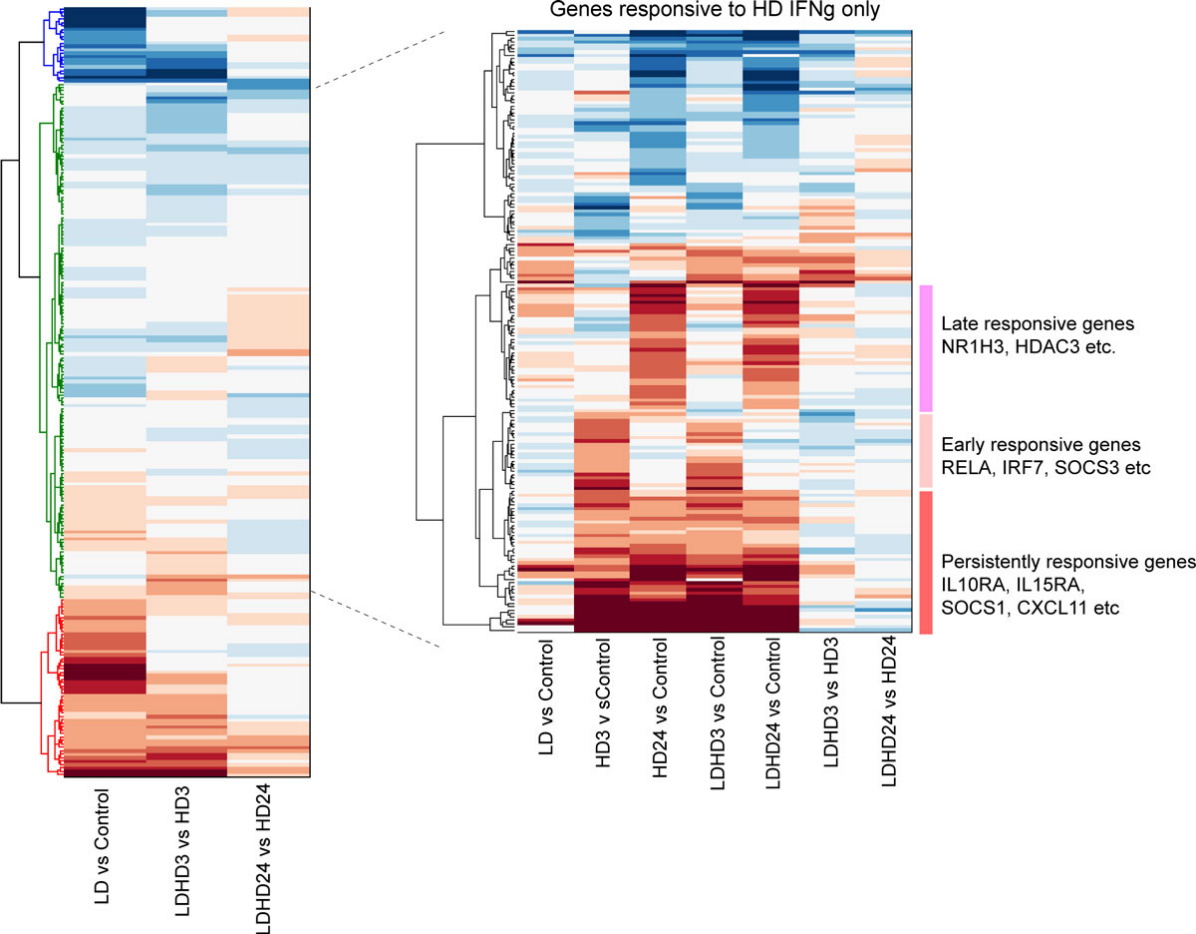
**A second step hierarchical clustering over the genes that only respond to HD**. Gene expression patterns are clustered according to the fold change under LD *vs *Control, HD *vs *Control (3hr), HD *vs *Control (24hr), LD+HD *vs *Control (3hr), LD+HD *vs *Control (24hr), LD+HD *vs *HD (3hr), and LD+HD *vs *HD (24hr). At least three major dynamical groups are identified among genes that are activated by HD stimulation.

Other genes that are not responsive to LD stimulation are further clustered according to the gene expression patterns. We found that a large portion of such genes can be activated by HD stimulation alone (Figure 5). Based on the guidance shown in Figure 3, they are potential candidates for the HD-responsive regulator in the three priming mechanisms. In addition, we found that these genes are activated with basically three dynamical patterns: early-, late-, and persistently-responsive dynamics (Figure 5). For example, RelA is found only expressed in the HD 3hr group, but not in the HD 24hr group, suggesting an early- dynamics. Suppressor of cytokine signaling 1 (SOCS1) is found in both HD 3hr and HD 24hr, indicating a persistent dynamics. This dynamical property is also necessary in assembling appropriate genes onto specific priming motifs.

Furthermore, five genes (SLC2A3, ST3GAL5, DNAJB1, STAT1, UBE2S) are identified as possible priming readout genes (x_3_), which show negligible expression under LD, but considerable higher expression under LD+HD than under HD alone. However, among the five genes, only UBE2S shows a significant change between LD+HD and HD (by ≥ 2 fold) that passes *t*-test with *p *< 0.05. Considering microarray data are usually noisy, one needs more quantitative measurements, e.g., real time PCR to confirm these results. Here we used the experimentally confirmed molecular species, such as phosphorylated STAT1 dimmer, IRF-1 and IP-10 as the priming readout [11]. After selecting and grouping genes based on the guideline in Figure 3, we then placed them in the context of regulatory networks in order to identify possible priming mechanism on the molecular interaction level. The regulatory network associated with these selected genes is constructed in IPA**^® ^**database (see Methods for details).

**Figure 6:**
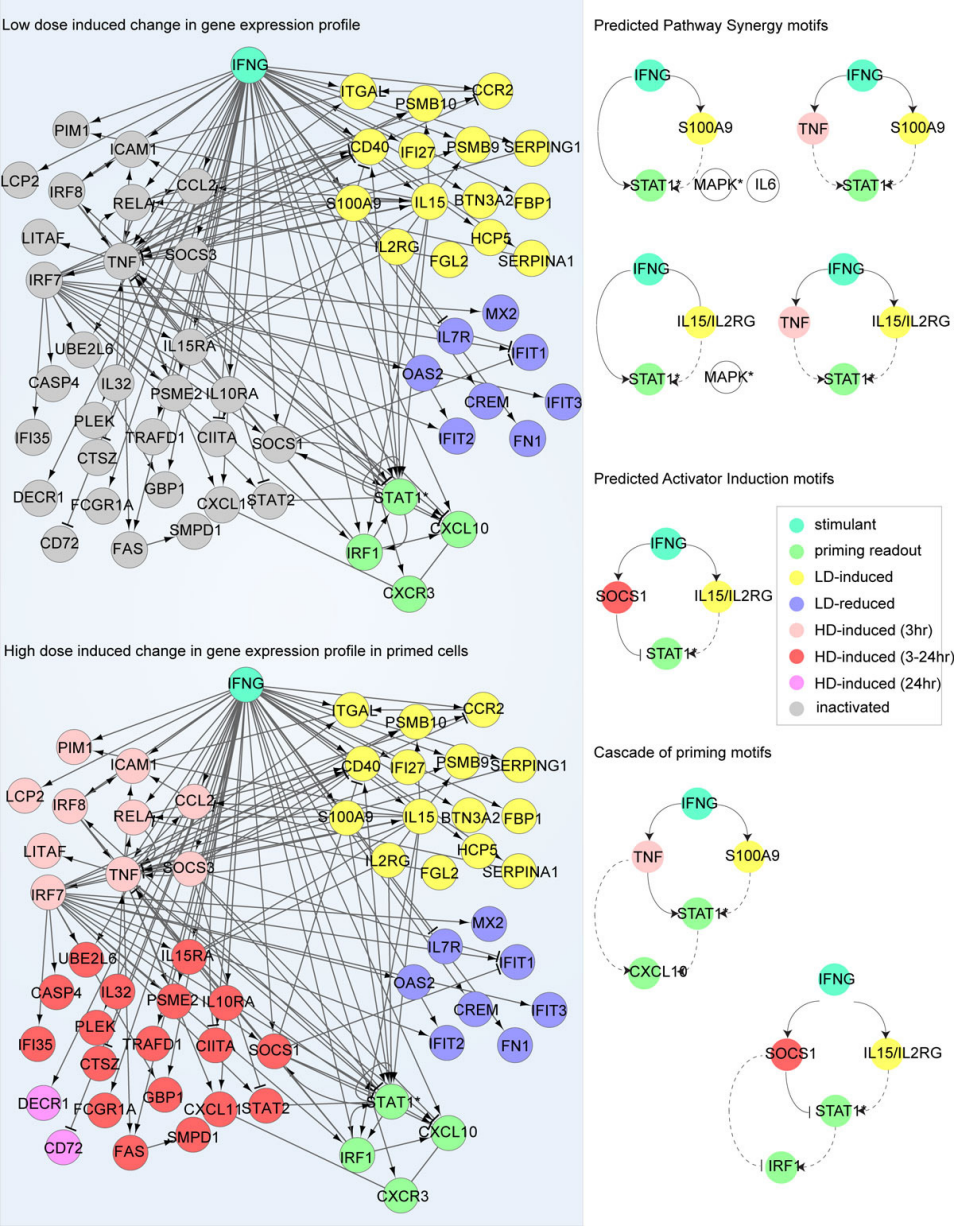
**Construction of the regulatory networks associated with the selected genes using IPA^® ^database**. Left panel: The selected groups of genes with different dose-response and dynamics are put into IPA**^® ^**database to identify signaling and regulatory relationships with the readout molecules (see Methods for details). Right panel: Priming motifs with different priming mechanisms are identified from the network under the guideline shown in Figure 3.

Here we show several potential PS and AI motifs identified from the regulatory network (Figure 6). For example, a PS motif (the second motif on the right) composes a HD-induced regulator (Tumor Necrosis Factor-α, TNFα), a LD-induced regulator (S100A9), and a readout (phosphorylated STAT1). The priming effect can be achieved by synergizing the two positive regulators, TNFα and S100A9, to get the STAT1 activity enhanced. This may be explained by the fact that an increased level of S100A9 by IFN-γ pretreatment may be able to activate P38 [27], which further up-regulates STAT1 activity. An alternative connection between S100A9 and STAT1 activity is through IL-6. S100A9 has been shown able to trigger interleukin 6 (IL-6) expression [28], which in turn stimulates STAT1. Therefore an autocrine signaling may also be involved. The true connection should be context dependent, and needs to be confirmed by further experiments. Moreover, a motif that fits in AI mechanism can also be identified from the regulatory network. This AI motif involves interleukin 15 (IL-15) and IL-2Rγ as the LD-responsive activator, and SOCS1 as the HD-inhibitor for STAT1 activity. It has been shown that both IL-15 and IL-2Rγ are able to increase STAT1 activity [29], and from the microarray analysis we show that they can be significantly induced by LD (> 2 fold, *p *< 0.05), while the inhibitory function of SOCS1 against STAT1 is only induced under HD. Therefore, the two counteractive pathways exert AI priming mechanism. As multiple priming motifs are identified on different levels in the regulatory network, we speculate these interconnected priming motifs may work in concert to induce an overall priming effect. A functional redundancy and robustness may also be achieved due to the complex cross-talks brought by these priming motifs in the regulatory network. As a matter of fact, both cascade and parallel layout priming motifs are found in this network (Figure 6). Detailed computational modeling can provide great help in understanding the potential functions, advantages and disadvantages brought forth by different combination of the priming motifs.

In our proposed strategy it is essential to examine the genes identified from the high throughput data in the context of the regulatory network. In many cases gene activities are correlated, e.g., due to a common upper stream regulator. As an illustrative example, suppose the activities of genes A and B are correlated and are both up-regulated by the low dose stimulant, but only A regulates the downstream readout gene C. Based on the absence of regulation from B to C in the regulatory network, one can only conclude that the existing experimental result suggests A, but not B, as a potential contributor to the priming of C. In another situation, if a molecular species (e.g., a transcription factor) shows priming effect, the priming effect may be transmitted to its downstream targets. The detailed model discussed later gives such an example.

### Functional clustering further suggest influence of low dose pretreatment on altering cellular functions

To investigate how LD priming affects macrophage cellular functions, we conducted the ontology analysis of the genes that show significant fold change (≥ 2 fold, *p* < 0.05) after LD priming. Additional file 2 shows the clustering result, and lists the top 10 significantly enriched molecular functions found for LD IFN-γ induced and reduced genes, respectively. We found that in general, genes that are significantly increased by LD priming are associated to inflammatory response and immune system process; genes that are significantly decreased are associated to negative regulation of T cell mediated cytotoxicity and immunity. This result suggests that LD priming prepares macrophages for a stronger inflammatory response by elevating a number of proinflammatory genes and inhibiting some negative regulators, reflecting a cellular adaptivity of innate immune cells.

### Low dose IFN-γ priming reprograms the gene expression profiles of macrophages

**Figure 7:**
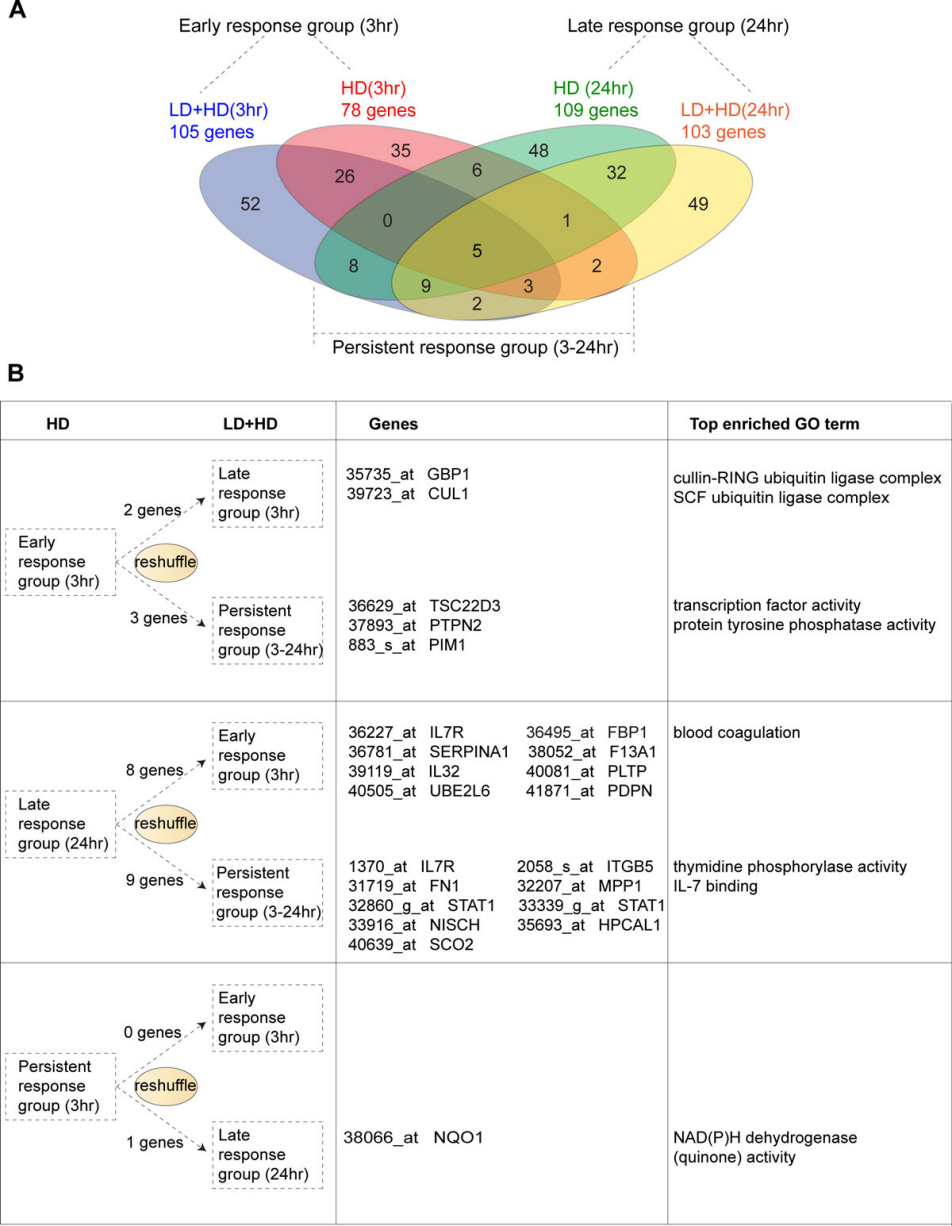
**The gene expression kinetics has been reprogrammed during the low dose IFN-γ priming**. (A) Four-way Venn Diagram demonstrated LD IFN-γ pretreatment reprogramed gene expression profiles of a large number of genes. (B) Kinetic reshuffling in a small number of genes is also identified. The gene name, probe name and the enriched gene ontology are shown in the second and the third column.

In order to find out whether LD IFN-γ pretreatment could possibly reprogram the gene expression dynamics, we grouped genes based on their induction dynamics under either HD or LD+HD stimulation (e.g., early-, late-, and persistent-response). As shown in Figure 7, we found that the number of early response genes increases in primed macrophages (from 78 to 105), while the number of late- and persistent-genes stays almost the same. Strikingly however, the actual composition of genes in each dynamical group has been changed by LD IFN-γ priming (Figure 7A). For example, nearly half of the genes from both the early- and the late-response groups are switched off (or to a statistically negligible level) in the primed cells (shown in the red ellipse and the green ellipse that does not overlap with others). Gene Ontology analysis shows that these genes are functionally associated with protein kinase inhibitor activity (the early-response group) and negative regulation of apoptosis (the late response group), indicating a functional change due to the LD pretreatment. Moreover, we also observed a reshuffling of genes among different dynamical groups (Figure 7B). For instance, five early-response genes are switched into either the late- or the persistent-response group, while 17 late-response genes are moved into the early- or the persistent-response group, in primed macrophages. Figure 7B lists the most significantly enriched gene ontologies associated to each group of these reshuffled genes. To sum up, the LD IFN-γ priming, to some extent if not globally, is able to reprogram the gene expression profile by switching genes on and off or changing their expression dynamics.

### Detailed experimental and model study further confirm the analysis result

We want to make it clear that the generic procedure shown in Figure 3 is not restricted to microarray data analysis. Microarray is a high throughput technique but less quantitative. One can only detect genes with significant fold change (usually by ≥ 2 fold). For many priming effects, the fold change is less than 2 [10,13]. Often more quantitative methods such as real time PCR are needed to confirm the microarray findings. Furthermore information on posttranslational and epigenetic modifications requires other techniques. In many applications, it is advantageous to combine time course data under LD, HD, and LD+HD stimulant obtained with different techniques. Here we use one example to illustrate this point.

Our microarray analysis suggested that STAT1 and SOCS1 may participate in a potential priming motif activated by IFN-γ (Figure 6), which is in consistence with the experimental investigations by Hu et al., [11]. Hu et al., reported that a pretreatment of a sub-threshold of IFN-γ sensitized the Janus kinase (JAK)-signal transducer and activator of transcription (STAT) signaling for a second dose of IFN-γ [11]. They found that a low dose IFN-γ exposure is able to switch on the transcription of STAT1. However, LD IFN-γ can only weakly activate the inhibitor SOCS1 in a transient manner [23]. Since STAT1 protein is more stable than SOCS1 protein, the elevated expression of STAT1 actually increased the pool for STAT1 docking and phosphorylation in response to the second dose of IFN-γ, thereby contributing to the induction of priming effect.

**Figure 8:**
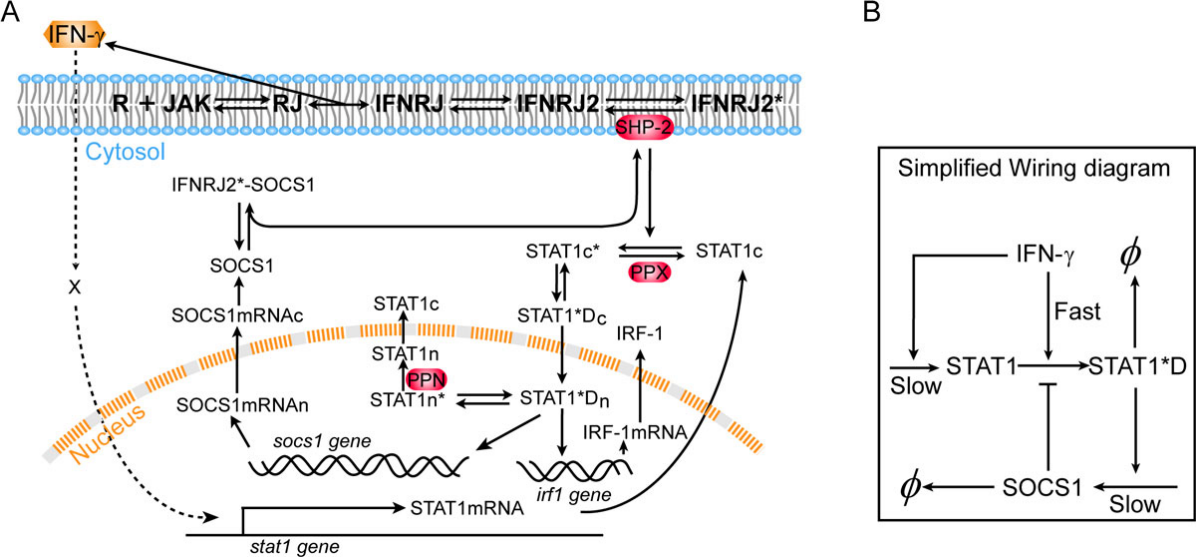
**Wiring diagram of IFN-γ signal transduction**. (A) Detailed wiring diagram used in the numerical simulations. IFN-γ binding to the cell membrane embedded Receptor/Jak complex leads to dimerization of the binding complex, and subsequent phosphorylation of Jak molecules; the phosphorylated dimer IFNRJ2* recruits and phosphorylates cytoplasmic STAT1 molecules; the latter dimerize and move into the nucleus, functioning as transcription factor to induce expressions of socs1, irf1, and many other genes; SOCS1 can either bind to Jak and inhibit its activity or compete with STAT1 on binding to IFNRJ2*. IFN-γ also induces Stat1 expression through an unknown mechanism independent of the Jak-STAT canonical pathway. Here we use "X" to represent an undetermined intermediate. For the molecular species, "c" and "n" refer to cytoplasm and nucleus, respectively; "*" refers to phosphorylation. This diagram is adapted from [31]. (B) A simplified wiring diagram to emphasize the three key processes with different time scales contributing to the priming effect.

To further analyze the mechanism, we performed computational analysis using ordinary differential equations (ODEs) model. The wiring diagram in Figure 8A summarizes the relevant biochemical events in the IFN-γ signaling pathway. A HD IFN-γ rapidly evokes Jak/STAT pathway, resulting in STAT1 phosphorylation and the expression of downstream genes, such as SOCS1, IRF-1 and IP-10 [11]. SOCS1 contains a kinase inhibitory region and Src homology 2 (SH2) domain [30]. It binds to Jak to inhibit its kinase activity, or alternatively it binds to IFN-γ receptor cytoplasmic docking sites as pseudo-substrates; in either way, SOCS1 functions in blocking STAT1 from phosphorylation [30]. The wiring diagram also includes the Jak/STAT independent induction of STAT1 expression by IFN-γ. Figure 8B also gives a simplified wiring diagram to show the processes of slow STAT1 synthesis, STAT1 activation through covalent modification, and inhibition from SOCS1 whose synthesis is activated by STAT1. The system dynamics is then modeled by ODEs [see Additional file 3 and 4 for details].

**Figure 9:**
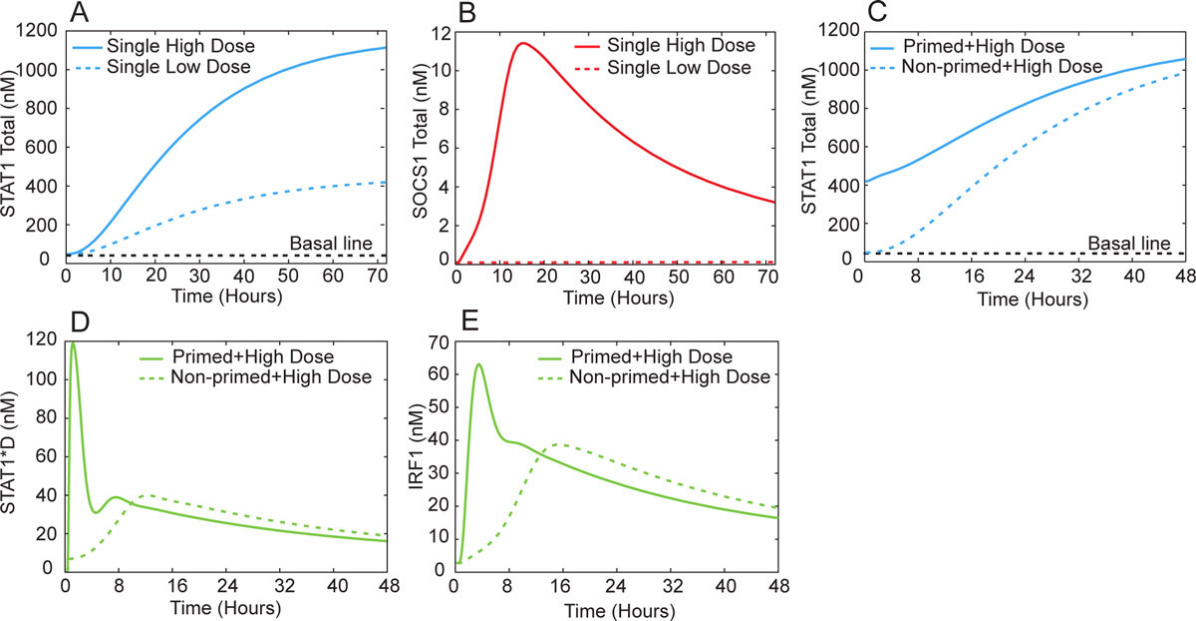
**Simulated time course of the IFN-γ signaling network**. (A, B) Macrophages are given 0.15µg/L and 5µg/L IFN-γ treatment for 72 hours, STAT1 responds quickly even at every low concentration while SOCS1 can only be turned on by high dose of IFN-γ; (C) Primed macrophages have an increased pool of STAT1 ready for phosphorylation and activation, while non-primed macrophages still take time to accumulate equal level of STAT1 (D); (D) Increased STAT1 in primed macrophages is rapidly activated by phosphorylation upon the second exposure to high concentration of IFN-γ; on the contrary, single high dose IFN-γ treatment of non-primed cells only initiates a slower and weaker STAT1 phosphorylation (D). The priming effect is also observed when Jak/STAT downstream genes are examined, including IRF-1(E).

Our computational analysis reveals a combination of the AI and PS mechanisms in this system. To illustrate, we see that under a 72 hour priming with LD IFN-γ (0.15 µg/L), the stimulated cells increase the expression of STAT1 but not SOCS1 (Figure 9A &9B); this is because LD priming does not turn on phosphorylation or activation of STAT1 which is required for SOCS1 production. However, the increased expression of STAT1 under LD pretreatment expands the pool of STAT1 for phosphorylation in response to the following HD IFN-γ (5 µg/L). Compared to protein binding/unbinding and covalent modifications such as phosphorylation, the gene expression process of STAT1 and SOCS1 is rather slow. Under a single HD, a fast Jak/STAT pathway signaling event quickly initializes SOCS1 gene expression, resulting in the suppression of STAT1 phosphorylation. For primed cells, however, the STAT1 gene expression dynamics is accelerated while that of SOCS1 remains unchanged. Before SOCS1 starts to function, the increased total STAT1 proteins and the STAT1 phosphorylation can add cooperatively, leading to a higher level of phosphorylated STAT1 dimer (STAT1*D) than that under single HD (Figure 9D). Figure 8B also suggests the combined AI/PS mechanism through the interplay among the three processes with different time scales. Our simulations suggest that the downstream genes such as IRF-1 also show priming effect (Figure 9E), which is in agreement with experimental observations [11].

Notice that in this model we only considered the coupling between IFN-γ induced STAT1 gene expression and the canonical Jak/STAT pathway. Figure 6 suggests a number of parallel pathways that may contribute to the observed IFN-γ priming effect. These pathways function together to make the temporal profile and amplitude of the priming phenomenon more complex.

## Conclusion

Molecules within a cell interact with each other and form a large interconnected network. Consequently cellular information seldom propagates linearly through a single pathway. The priming effect, which widely studied using immune cells, is such an example. Based on our previous *in silico *studies [25], in this work we proposed a generic procedure to identify possible molecular candidates contributing to the priming effect through combined experimental time course measurement, subsequent data analysis and computational modeling. We demonstrated the procedure with high throughput microarray and other data on interferon-γ induced priming effects. This procedure is generally applicable to other similar problems. Especially it is of great significance to examine the generality and the specificity of the observed priming effects, in terms of stimulant and cell types. One may perform systematic screening using the proposed procedure combining with high throughput measurements, at both transcriptome and proteome levels.

## Methods

### Microarray data processing

The microarray data were downloaded from Gene Expression Omnibus (GEO, accession number: GDS1365). The data record the expression profile of approximately 12,000 gene probes with 3 independent pools. This is the only dataset we could find from GEO that include systematic time course measurement under either single dose or sequential stimulations (Control, HD 3hr, HD 24hr, LD Control, LD+HD 3hr, LD+HD 24hr. LD: 0.15 µg/L IFN-γ, HD: 5 µg/L IFN-γ).

In order to analyze the gene expression pattern, we first filtered out genes that contain no "Present Call" in all three independent pools. Genes without differential expression (by fold change < 2) under all of the following conditions were also filtered out: LD *vs *Control, HD (3hr) *vs *Control, HD (24hr) *vs *Control, LD+HD (3hr) *vs *Control and LD+HD (24hr) *vs *Control. All Differential expression was statistically analyzed by Welch's *t*-test with FDR correction. The threshold of *p*-value is set to be 0.05.

### Network construction with the IPA database

We used the commercial database IPA^® ^(@Ingenuity) to query the molecular interactions among interested genes and products. IPA^® ^assembles the signaling/regulatory network on a literature basis. Database query was restricted to immune cells and immune cell lines in *Mus musculus *or *Homo sapiens*. Interaction type was chosen to be either direct or indirect (i.e., interaction with intermediates). Prediction on potential priming candidates was made by comparing the priming motifs shown in Figure 2 and the signaling/regulatory networks constructed by IPA^®^.

### Detailed modeling with ordinary differential equations

We used a mathematic model adapted from Yamada et al. [31] to simulate the dynamics of Jak/STAT pathway in macrophages under different stimulation scenarios. Hu et al. have reported that increased expression of STAT1 induced by the first dose of IFN-γ treatment was responsible for sensitization of Jak/STAT1 pathway [11], we therefore added two additional reactions to the original model: STAT1 transcription triggered by IFN-γ and STAT1 translation. In addition, we introduced two reactions describing IRF-1 transcription and translation. Adding these two reactions allows us to exam the expression behavior of downstream gene IRF-1 for priming effects. As it is unclear how IFN-γ affects STAT1 expression, we proposed that an unknown intermediate × transduces the signal from IFN-γ to STAT1 gene.

As shown in additional file 3 and 4, our model includes 36 variables and 50 parameters. Most of the rate equations are presented using Mass-action kinetics. Several equations presenting gene transcription are denoted using Michaelis-Menten kinetics. We employed the same initial conditions for Jak, IFN-γ receptor, PPX, PPN and SHP-2 as in the work of Yamada et al. Other initial conditions are set to be the steady-state values achieved given zero IFN-γ signal. These ODEs are solved using standard ODE solver in Matlab. In our simulation, macrophages were primed with 0.15 µg/L of IFN-γ for 3 days, after which cells were washed for 10 minutes with fresh medium and re-stimulated with 5 µg/L IFN-γ for 2 days [11]. The total STAT1 and SOCS1 proteins under repetitive two stimulations and single high dose of IFN-γ treatment were analyzed. In addition, phosphorylated STAT1 dimer and IRF-1 were examined as readouts to quantify the level of priming effect [31].

## Abbreviations

LPS: lipopolysaccharide; IFN-γ: interferon-gamma; Jak: Janus kinase; STAT1: signal transducer and activator of transcription 1; IRF-1: interferon regulatory factor 1; IP-10: interferon gamma-induced protein 10; TLR4: Toll-like receptor 4; LD: low dose; HD: high dose; AI: activator induction; PS: pathway synergy; SD: suppressor deactivation; SOCS1: suppressor of cytokine signaling 1; TNFα: tumor necrosis factor-alpha; IL-6: interleukin-6; IL-15: interleukin-15; SH2: Src homology 2; STAT1*D: phosphorylated STAT1 dimer; PPX: unidentified phosphatase in the cytoplasm; PPN: nuclear phosphatase; SHP-2: SH2 domain-containing tyrosine phosphatase 2; IFNR: interferon-γ receptor; RJ: IFNR-Jak complex; IFNRJ: IFN-γ-IFNR-Jak complex; IFNRJ2: IFN-γ-IFNR-Jak complex dimer; IFNRJ2*: IFN-γ-IFNR-Jak complex phosphorylated dimer; STAT1c: cytoplasmic STAT1; STAT1n: nuclear STAT1; STAT1c*: phosphorylated cytoplasmic STAT1; STAT1n*: phosphorylated nuclear STAT1; STAT1n*Dn: phosphorylated nuclear STAT1 dimer; STAT1n*Dc: phosphorylated cytoplasmic STAT1 dimer.

## Competing interests

The authors declare that they have no competing interests.

## Authors' contributions

JX designed the project; YF, XJ, HZ and JX performed computational modeling; YF, XJ and JX wrote the paper.

## Supplementary Material

Additional file 1 **The maximum change distribution of regulators induced by HD or LD+HD under each priming mechanism**. First column: Sample distribution in term of maximum change of x_1 _or x_2 _under HD alone (i.e., $\underset{\text{max}}{\Delta }x_{i,HD}$). Second column: distribution of changes between the maximum induction under LD+HD and the maximum induction under HD alone (i.e., $\underset{\text{max}}{\Delta }x_{i,LD+HD}-\underset{\text{max}}{\Delta }x_{i,HD}$). For PS and AI, there is a great increase in x_1 _under HD, but the maximum expression of x_1 _under LD+HD and HD alone shows no significant difference; Similarly for PS, x_2 _expression is enhanced by HD, whereas maximum expression of x_2 _under LD+HD is almost the same with that under HD alone. Click here for file

Additional file 2 **Functional clustering of genes significant increased or decreased (≥2 fold) under LD IFN-γ**. The functional clustering is computed according to the enrichment of gene ontology retrieved from GOStat database. The top 10 significantly physiological functions of either LD-induced or LD-reduced genes are listed on the right. The functional clustering is computed by Cytoscape pluggin BiNGO 2.44. Click here for file

Additional file 3 **Biochemical reactions and parameters for the computational model**. Click here for file

Additional file 4 **Variables and ordinary differentiation equations of the computational model**. Click here for file
