# Supplementary figures and images for: A strategy to study pathway cross-talks of cells under repetitive exposure to stimuli
Source: BMC Syst Biol. 2012 Dec 17;6(Suppl 3):S6. doi: 10.1186/1752-0509-6-S3-S6 (PMC3524319; doi:10.1186/1752-0509-6-S3-S6)

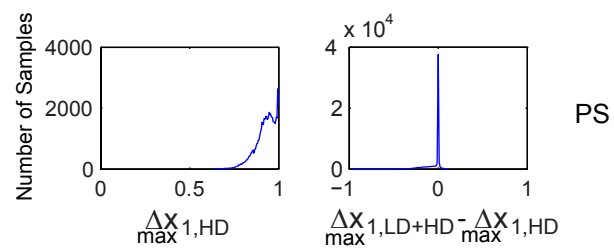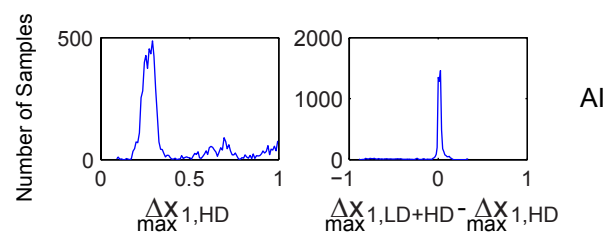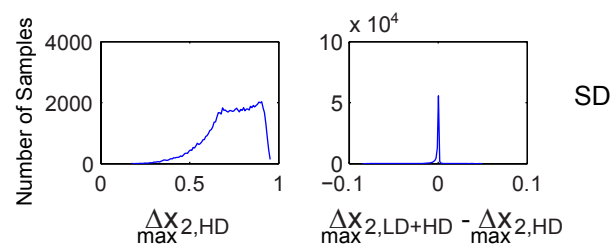

Supplement: Additional file 1 — The maximum change distribution of regulators induced by HD or LD+HD under each priming mechanism. First column: Sample distribution in term of maximum change of x1 or x2 under HD alone (i.e., Δmaxxi,HD). Second column: distribution of changes between the maximum induction under LD+HD and the maximum induction under HD alone (i.e., Δmaxxi,LD+HD-Δmaxxi,HD). For PS and AI, there is a great increase in x1 under HD, but the maximum expression of x1 under LD+HD and HD alone shows no significant difference; Similarly for PS, x2 expression is enhanced by HD, whereas maximum expression of x2 under LD+HD is almost the same with that under HD alone. [file 1752-0509-6-S3-S6-S1.pdf]
