# Supplementary material for: A strategy to study pathway cross-talks of cells under repetitive exposure to stimuli
Source: BMC Syst Biol. 2012 Dec 17;6(Suppl 3):S6. doi: 10.1186/1752-0509-6-S3-S6 (PMC3524319; doi:10.1186/1752-0509-6-S3-S6)
Supplement: Additional file 2 — Functional clustering of genes significant increased or decreased (≥2 fold) under LD IFN-γ. The functional clustering is computed according to the enrichment of gene ontology retrieved from GOStat database. The top 10 significantly physiological functions of either LD-induced or LD-reduced genes are listed on the right. The functional clustering is computed by Cytoscape pluggin BiNGO 2.44. [file 1752-0509-6-S3-S6-S2.pdf]

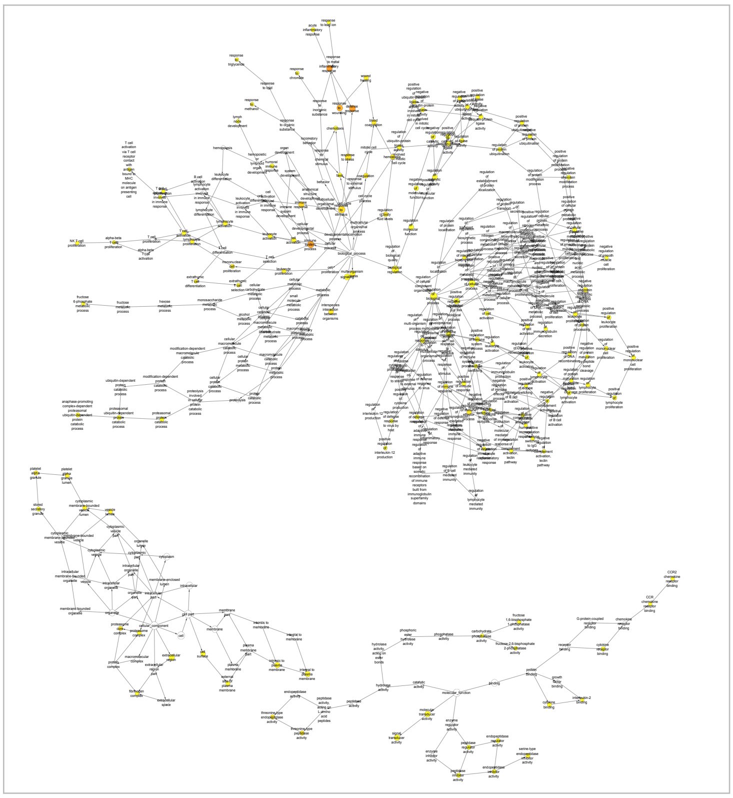

Low dose induced cellular function (top 10)

inflammatory response
defense response
immune system process
response to wounding
immune response
response to stimulus
response to stress
blood coagulation
coagulation
regulation of immune effector process

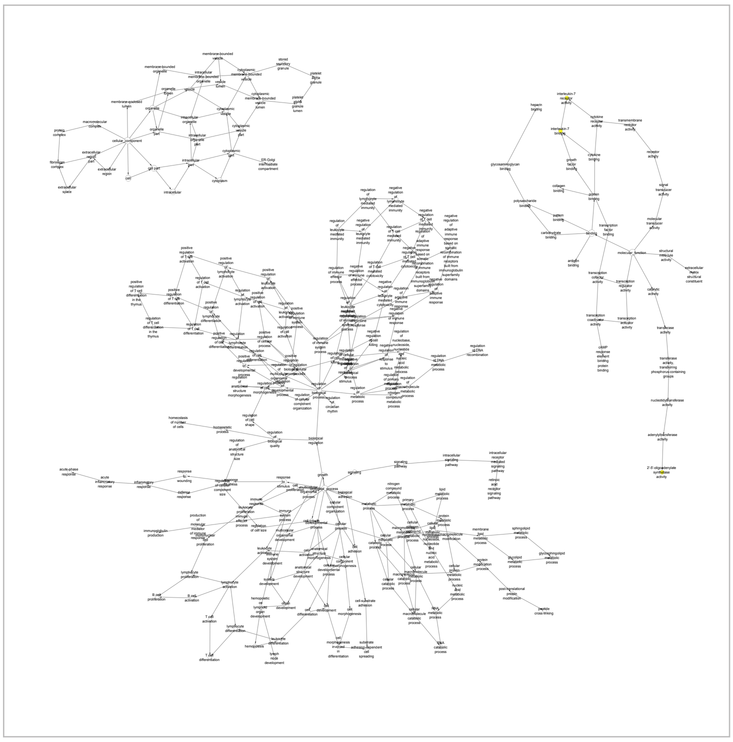

Low dose reduced cellular function (top 10)

IL7 receptor activity
IL7 binding
2'-5'-oligoadenylate synthetase activity
negative regulation of T cell mediated cytotoxicity
positive regulation of T cell differentitation in the thymus
cAMP repsonse element binding protein binding
negative regulation of leukocyte mediated cytotoxicity
negative regulation of T cell mediated immunity
negative regulation of cell killing
fibrinogen complex
