# Supplementary material for: A strategy to study pathway cross-talks of cells under repetitive exposure to stimuli
Source: BMC Syst Biol. 2012 Dec 17;6(Suppl 3):S6. doi: 10.1186/1752-0509-6-S3-S6 (PMC3524319; doi:10.1186/1752-0509-6-S3-S6)
Supplement: Additional file 3 — Biochemical reactions and parameters for the computational model. [file 1752-0509-6-S3-S6-S3.pdf]

Additional file 3 Biochemical reactions and Parameters for the computational model (adapted from [1])

Biochemical reactions	Parameters	Notes
[R]+[JAK]↔[RJ]	k1=100	
	k_1=0.05	
[IFN-γ]+[RJ] ↔ [IFNRJ]	k2=20	
	k_2=0.02	
2[IFNRJ] ↔ [IFNRJ2]	k3=40	
	k_3=0.2	
[IFNRJ2]→[IFNRJ2*]	k4=0.005	
[IFNRJ2*]+[STAT1c] ↔ [IFNRJ2*-STAT1c]	k5=8	
	k_5=0.8	
[IFNRJ2*-STAT1c]→[IFNRJ2*]+[STAT1c*]	k6=0.4	
[IFNRJ2*]+[STAT1c*]↔[IFNRJ2*-STAT1c*]	k7=5	
	k_7=0.5	
2[STAT1c*]↔[STAT1c*-STAT1c*]	k8=20	
	k_8=0.1	
[IFNRJ2*]+[SHP-2] ↔[IFNRJ2*-SHP-2]	k9=1	
	k_9=0.2	
[IFNRJ2*-SHP-2]→[IFNRJ2]+[SHP-2]	k10=0.003	
[PPX]+[STAT1c*]↔[PPX-STAT1c*]	k11=1	
	k_11=0.2	
[PPX-STAT1c*]→[PPX]+[STAT1c]	k12=0.003	Adapted from Yamada's model [1].
[PPX]+[STAT1c*-STAT1c*]↔[PPX-STAT1c*-STAT1c*]	k11=1	
	k_11=0.2	
[PPX-STAT1c*-STAT1c*]→[PPX]+[STAT1c-STAT1c*]	k12=0.003	
[STAT1c]+[STAT1c*]↔[STAT1c-STAT1c*]	k13=0.0002	
	k_13=0.2	
[STAT1c*-STAT1c*]→[STAT1n*-STAT1n*]	k14 =0.005	
2[STAT1n*]↔[STAT1n*-STAT1n*]	k7=5	
	k_7=0.5	
[PPN]+[STAT1n*]↔[PPN-STAT1n*]	k15 =1	
	k_15 = 0.2	
[PPN-STAT1n*]→[PPN]+[STAT1n]	k16 =0.005	
[PPN]+[STAT1n*-STAT1n*]↔[PPN-STAT1n*-STAT1n*]	k15 =1	
	k_15 = 0.2	
[PPN-STAT1n*-STAT1n*]→[PPN]+[STAT1n-STAT1n*]	k16 =0.005	
[STAT1n]+[STAT1n*]↔[STAT1n-STAT1n*]	k13=0.0002	
	k_13=0.2	
[STAT1n]→[STAT1c]	k17 =0.05	
d[SOCS1_mRNAn]/dt =k18+k18a[STAT1n*-STAT1n*]/(k18b+[STAT1n*-STAT1n*])	k18=1×10^{-9}	Basal transcription rate of SOCS mRNA, fitted.
	k18a = 0.002 nM/s	Maximal SOCS1 mRNA transcription rate activated by STAT1, fitted.
	k18b = 400 nM	
[SOCS1_mRNAn]→[SOCS1_mRNAc]	k19 = 0.001	
d[SOCS1]/dt = k20[SOCS1_mRNAc]	k20 = 0.01	Adapted from Yamada's model [1].
[SOCS1]+[IFNRJ2*]↔[SOCS1-IFNRJ2*]	k21=20	
	k_21=0.1	

Additional file 3 (Cont'd)

Biochemical reactions	Parameters	Notes
d[SOCS1_mRNAc]/dt= -k22[SOCS1_mRNAc]	k22 =0.0008	SOCS1 mRNA degradation rate, fitted.
d[SOCS1]/dt= -k23[SOCS1]	k23 =0.00013	SOCS1 has a half-life of 1.5 hours [2].
[STAT1c]+[SOCS1-IFNRJ2*]↔[SOCS1-IFNRJ2*-STAT1c]	k5=8	Adapted from Yamada's model [1].
	k_5=0.8	
[SHP-2]+[SOCS1-IFNRJ2*-STAT1c]↔[SOCS1-IFNRJ2*-STAT1c-SHP-2]	k9=1	
	k_9=0.2	
[SOCS1-IFNRJ2*-STAT1c-SHP-2]→[SOCS1]+[IFNRJ2]+[STAT1c]+[SHP-2]	k10=0.003	
[SOCS1-IFNRJ2*-STAT1c-SHP-2]→[IFNRJ2*-STAT1c-SHP-2]	k23 =0.00013	SOCS1 has a half-life of 1.5 hours [2].
d[X]/dt=k24a[IFN-γ]/(k24b+[IFN-γ])-k_24[X]	k24a=5×10^{-6} nM/s	Maximal X expression rate stimulated by signal, fitted.
	k24b=0.016 nM	Michaelis-Menten constant, fitted.
	k_24=0.0002	X degradation rate, fitted.
d[STAT1_mRNA]/dt=k25+k25a[X]/(k25b+[X]) -k_25[STAT1_mRNA]	k25=2×10^{-9}	STAT1 basal transcription rate, fitted.
	k25a=8×10-4 nM/s	Maximal STAT1 transcription rate stimulated by X, fitted.
	k25b=0.4 nM	Michaelis-Menten constant, fitted.
	k_25=0.000035	STAT1 mRNA has a half-life of about 7.4 hours [3].
d[STAT1]/dt=k26[STAT1_mRNA]-k_26[STAT1]	k26=0.01	STAT1 translation rate, fitted.
	k_26=0.000012	STAT1 has a half-life of 16 hours[2].
d[IRF1_mRNA/dt=k27a[STAT1n*-STAT1n*]/(k27b+[STAT1n*-STAT1n*])-k_27[IRF1_mRNA]	k27a=0.004 nM/s	Fitted
	k27b=400	Fitted
	k_27=0.00016	IRF1 mRNA has a half-life of 1.2 hours[3].
d[IRF1]/dt=k28[IRF1_mRNA]-k_28[IRF1]	k28=0.01	Fitted
	k_28=0.00038	IRF1 has a half-life of about 30 minutes[4].

*Initial condition: [R] =12nM, [Jak]=12nM, [SHP-2]=100nM, [PPX]=50nM and [PPN]=60nM

* The first and second order rate constants are represented in units of $second^{-1}$ and 10^{6} $molar^{-1}$ $second^{-1}$, respectively.

Reference

1. Yamada S, Shiono S, Joo A, Yoshimura A: **Control mechanism of JAK/STAT signal transduction pathway.** *FEBS Lett* 2003, **534:**190-196.
2. Siewert E, Muller-Esterl W, Starr R, Heinrich PC, Schaper F: **Different protein turnover of interleukin-6-type cytokine signalling components.** *Eur J Biochem* 1999, **265:**251-257.

3. Sharova LV, Sharov AA, Nedorezov T, Piao Y, Shaik N, Ko MSH: **Database for mRNA Half-Life of 19 977 Genes Obtained by DNA Microarray Analysis of Pluripotent and Differentiating Mouse Embryonic Stem Cells.** *DNA Research* 2009, **16:**45-58.

4. Watanabe N, Sakakibara J, Hovanessian AG, Taniguchi T, Fujita T: **Activation of IFN-β element by IRF-1 requires a post-translational event in addition to IRF-1 synthesis.** *Nucleic Acids Res* 1991, **19:**4421-4428.
