# Supplementary material for: A strategy to study pathway cross-talks of cells under repetitive exposure to stimuli
Source: BMC Syst Biol. 2012 Dec 17;6(Suppl 3):S6. doi: 10.1186/1752-0509-6-S3-S6 (PMC3524319; doi:10.1186/1752-0509-6-S3-S6)
Supplement: Additional file 4 — Variables and ordinary differentiation equations of the computational model. [file 1752-0509-6-S3-S6-S4.pdf]

Additional file 4 Variables and Ordinary Differentiation Equations of the Computational Model

Variable Index	Gene-Symbol	Differentiation equation
S	IFN-γ	
x_1	R (IFN-γ Receptor)	$dx_1/dt = -k_1 \cdot x_1 \cdot x_2 + k_{-1} \cdot x_3$
x_2	JAK	$dx_2/dt = -k_1 \cdot x_1 \cdot x_2 + k_{-1} \cdot x_3$
x_3	RJ	$dx_3/dt = k_1 \cdot x_1 \cdot x_2 - k_{-1} \cdot x_3 - k_2 \cdot S \cdot x_3 + k_{-2} \cdot x_4$
x_4	IFNRJ	$dx_4/dt = k_2 \cdot S \cdot x_3 - k_{-2} \cdot x_4 - 2 \cdot k_3 \cdot x_4 \cdot x_4 + 2 \cdot k_{-3} \cdot x_5$
x_5	IFNRJ2	$dx_5/dt = k_3 \cdot x_4 \cdot x_4 - k_{-3} \cdot x_5 - k_4 \cdot x_5 + k_{10} \cdot x_{13} + k_{10} \cdot x_{31}$
x_6	IFNRJ2*	$dx_6/dt = k_4 \cdot x_5 - k_5 \cdot x_6 \cdot x_7 + k_{-5} \cdot x_8 + k_6 \cdot x_8 - k_7 \cdot x_6 \cdot x_9 + k_{-7} \cdot x_{10}$
		$-k_9 \cdot x_6 \cdot x_{12} + k_{-9} \cdot x_{13} - k_{21} \cdot x_{28} \cdot x_6 + k_{-21} \cdot x_{29}$
x_7	STAT1c	$dx_7/dt = -k_5 \cdot x_6 \cdot x_7 + k_{-5} \cdot x_8 + k_{12} \cdot x_{15} - k_{13} \cdot x_7 \cdot x_9 + k_{-13} \cdot x_{17} + k_{17} \cdot x_{25}$
		$-k_5 \cdot x_7 \cdot x_{29} + k_{-5} \cdot x_{30} + k_{10} \cdot x_{31} + k_{26} \cdot x_{33} - k_{-26} \cdot x_7$
x_8	IFNRJ2*-STAT1c	$dx_8/dt = k_5 \cdot x_6 \cdot x_7 - k_{-5} \cdot x_8 - k_6 \cdot x_8$
x_9	STAT1c*	$dx_9/dt = k_6 \cdot x_8 - k_7 \cdot x_6 \cdot x_9 + k_{-7} \cdot x_{10} - 2 \cdot k_8 \cdot x_9 \cdot x_9 + 2 \cdot k_{-8} \cdot x_{11}$
		$-k_{11} \cdot x_{14} \cdot x_9 + k_{-11} \cdot x_{15} - k_{13} \cdot x_7 \cdot x_9 + k_{-13} \cdot x_{17}$
x_{10}	IFNRJ2*-STAT1c*	$dx_{10}/dt = k_7 \cdot x_6 \cdot x_9 - k_{-7} \cdot x_{10}$
x_{11}	STAT1c*-STAT1c*	$dx_{11}/dt = k_8 \cdot x_9 \cdot x_9 - k_{-8} \cdot x_{11} - k_{14} \cdot x_{11} - k_{11} \cdot x_{14} \cdot x_{11} + k_{-11} \cdot x_{16}$
x_{12}	SHP-2	$dx_{12}/dt = -k_9 \cdot x_6 \cdot x_{12} + k_{-9} \cdot x_{13} + k_{10} \cdot x_{13} - k_9 \cdot x_{12} \cdot x_{30} + k_{-9} \cdot x_{31} + k_{10} \cdot x_{31}$
x_{13}	IFNRJ2*-SHP-2	$dx_{13}/dt = k_9 \cdot x_6 \cdot x_{12} - k_{-9} \cdot x_{13} - k_{10} \cdot x_{13}$
x_{14}	PPX	$dx_{14}/dt = -k_{11} \cdot x_{14} \cdot x_9 + k_{-11} \cdot x_{15} + k_{12} \cdot x_{15} - k_{11} \cdot x_{14} \cdot x_{11} + k_{-11} \cdot x_{16} + k_{12} \cdot x_{16}$
x_{15}	PPX-STAT1c*	$dx_{15}/dt = k_{11} \cdot x_{14} \cdot x_9 - x_{-11} \cdot x_{15} - k_{12} \cdot x_{15}$
x_{16}	PPX-STAT1c*-STAT1c*	$dx_{16}/dt = k_{11} \cdot x_{14} \cdot x_{11} - k_{-11} \cdot x_{16} - k_{12} \cdot x_{16}$
x_{17}	STAT1c-STAT1c*	$dx_{17}/dt = k_{12} \cdot x_{16} + k_{13} \cdot x_7 \cdot x_9 - k_{-13} \cdot x_{17}$
x_{18}	STAT1n*	$dx_{18}/dt = -2 \cdot k_7 \cdot x_{18} \cdot x_{18} + 2 \cdot k_{-7} \cdot x_{19} - k_{15} \cdot x_{20} \cdot x_{18} + k_{-15} \cdot x_{21} - k_{13} \cdot x_{25} \cdot x_{18}$
		$+k_{-13} \cdot x_{24}$
x_{19}	STAT1n*-STAT1n*	$dx_{19}/dt = k_{14} \cdot x_{11} + k_7 \cdot x_{18} \cdot x_{18} - k_{-7} \cdot x_{19} - k_{15} \cdot x_{20} \cdot x_{19} + k_{-15} \cdot x_{22}$
x_{20}	PPN	$dx_{20}/dt = -k_{15} \cdot x_{20} \cdot x_{18} + k_{-15} \cdot x_{21} + k_{16} \cdot x_{21} - k_{15} \cdot x_{20} \cdot x_{19} + k_{-15} \cdot x_{22} + k_{16} \cdot x_{22}$
x_{21}	PPN-STAT1n*	$dx_{21}/dt = k_{15} \cdot x_{20} \cdot x_{18} - k_{-15} \cdot x_{21} - k_{16} \cdot x_{21}$
x_{22}	PPN-STAT1n*-STAT1n*	$dx_{22}/dt = k_{15} \cdot x_{20} \cdot x_{19} - k_{-15} \cdot x_{22} - k_{16} \cdot x_{22}$
x_{23}	STAT1n-STAT1n	$dx_{23}/dt = 0$
x_{24}	STAT1n-STAT1n*	$dx_{24}/dt = k_{16} \cdot x_{22} + k_{13} \cdot x_{25} \cdot x_{18} - k_{-13} \cdot x_{24}$
x_{25}	STAT1n	$dx_{25}/dt = -k_{13} \cdot x_{25} \cdot x_{18} + k_{-13} \cdot x_{24} + k_{16} \cdot x_{21} - k_{17} \cdot x_{25}$
x_{26}	SOCS1_mRNAn	$dx_{26}/dt = k_{18} + k_{18a} \cdot x_{19} / (k_{18b} + x_{19}) - k_{19} \cdot x_{26}$
x_{27}	SOCS1_mRNAc	$dx_{27}/dt = k_{19} \cdot x_{26} - k_{22} \cdot x_{27}$

x_{28}	SOCS1	$dx_{28}/dt = k_{20} \cdot x_{27} - k_{21} \cdot x_{28} \cdot x_6 + k_{-21} \cdot x_{29} - k_{23} \cdot x_{28} + k_{10} \cdot x_{31}$
x_{29}	SOCS1-IFNRJ2*	$dx_{29}/dt = k_{21} \cdot x_{28} \cdot x_6 - k_{-21} \cdot x_{29} - k_5 \cdot x_7 \cdot x_{29} + k_{-5} \cdot x_{30}$
x_{30}	SOCS1-IFNRJ2*-STAT1c	$dx_{30}/dt = k_5 \cdot x_7 \cdot x_{29} - k_{-5} \cdot x_{30} - k_9 \cdot x_{12} \cdot x_{30} + k_{-9} \cdot x_{31}$
x_{31}	SOCS1-IFNRJ2*-STAT1c-SHP-2	$dx_{31}/dt = k_9 \cdot x_{12} \cdot x_{30} - k_{-9} \cdot x_{31} - k_{10} \cdot x_{31} - k_{23} \cdot x_{31}$
x_{32}	IFNRJ2*-STAT1c-SHP-2	$dx_{32}/dt = k_{23} \cdot x_{31}$
x_{33}	STAT1c_mRNA	$dx_{33}/dt = k_{25} + k_{25a} \cdot x_{36} / (k_{25b} + x_{36}) - k_{-25} \cdot x_{33}$
x_{34}	IRF1-mRNA	$dx_{34}/dt = k_{27a} \cdot x_{19} / (k_{27b} + x_{19}) - k_{-27} \cdot x_{34}$
x_{35}	IRF1	$dx_{35}/dt = k_{28} \cdot x_{34} - k_{-28} \cdot x_{35}$
x_{36}	X	$dx_{36}/dt = k_{24a} \cdot S / (k_{24b} + S) - k_{-24} \cdot x_{36}$
